# Impact of moment arm on torque production of the knee extensors in children

**DOI:** 10.14814/phy2.14521

**Published:** 2020-09-01

**Authors:** Nobuaki Tottori, Tadashi Suga, Miyuki Hori, Tadao Isaka, Satoshi Fujita

**Affiliations:** ^1^ Faculty of Sport and Health Science Ritsumeikan University Kusatsu Japan; ^2^ Research Organization of Science and Technology Ritsumeikan University Kusatsu Japan

**Keywords:** isometric/isokinetic torque, magnetic resonance imaging, quadriceps femoris muscle volume, torque‐producing capacity

## Abstract

The joint moment arm (MA) dimension is related to joint torque in adults. However, this relationship remains unexplored in children. In this study, we aimed to determine the relationship between MA and joint torque of the knee extensors in this young population. The quadriceps femoris muscle volume (MV) and knee extensor MA in 20 preadolescent boys (age: 10.7 ± 0.9 years) were measured using magnetic resonance imaging. The knee extensor isometric and isokinetic torques were measured using a dynamometer. The isokinetic torque measurements were performed using slow and fast angular velocities at 60°/s and 180°/s respectively. The knee extensor torque‐producing capacities were assessed as the knee extensor isometric or isokinetic torque per the quadriceps femoris MV. The quadriceps femoris MV correlated significantly with all three knee extensor isometric and isokinetic torques (*r* = 0.513–0.804, *p* < .05 for all). The knee extensor MA also correlated significantly with the three knee extensor isometric and isokinetic torques (*r* = 0.701–0.806, *p* ≤ .001 for all). Furthermore, the knee extensor MA correlated significantly with all three knee extensor torque‐producing capacities (*r* = 0.488–0.701, *p* < .05 for all). These findings suggest that in addition to adults, greater MA plays an important role in achieving higher joint torque production of the knee extensors in preadolescent boys. This study is the first to determine the impact of MA dimension on joint torque production in children.

## Graphical abstract

Impact of moment arm dimension on joint torque production remains unexplored in children. Moment arm correlated with joint torque productions during isometric and isokinetic contractions of the knee extensors in this population. Moment arm dimension plays an important role in achieving superior torque production in children.

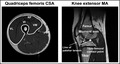

## 1 INTRODUCTION

The joint torque, which is assessed as muscle strength, plays an important role in various situations throughout the lifetime. In particular, the magnitude of joint torque is associated with superior sport performances in various athletes (Alexander, 1989; Hoshikawa et al., 2009; Sliwowski, Grygorowicz, Wieczorek, & Jadczak, 2017) and can improve the quantity and quality of daily life activities in children and adults (Faigenbaum et al., 2009; Newman et al., 2006; Ruiz et al., 2008; Strong et al., 2005). The joint torque is determined by agonist muscle size (Fukunaga et al., 2001). Many previous studies reported a strong relationship between joint torque and muscle size in adults (Baxter & Piazza, 2014; Blazevich, Coleman, Horne, & Cannavan, 2009; Fukunaga et al., 2001; Trezise, Collier, & Blazevich, 2016). By contrast, such a relationship is poorly understood in children.

Theoretically, joint torque is not only determined by muscle size but also by moment arm (MA). Blazevich et al. (2009) reported a positive relationship between MA dimension and isometric torque of the knee extensor in adults. Furthermore, Baxter and Piazza (2014) reported that MA dimension correlated with both isometric and isokinetic torques of the plantar flexors in adults. However, to the best of our knowledge, no study has examined the relationship between MA dimension and joint torque in children. O'Brien, Reeves, Baltzopoulos, Jones, and Maganaris (2010) reported that a relative difference of the knee extensor MA between preadolescent children and adults was lower than that of the quadriceps femoris MV (e.g., 36 and 190%, respectively, between boys and men); in other words, morphological maturation may be lower for muscle size than for joint dimension in preadolescent children. In addition, previous studies reported that neural activation level during muscle contraction may be lower in preadolescent children than in adults (Kluka et al., 2015; O'Brien et al., 2010). As a result of smaller muscle size and lower muscle neural activity for children than those for adults, the contribution of MA dimension to joint torque may be greater in children than in adults. Therefore, we hypothesized that greater MA would relate to higher joint torque in children, especially preadolescent children.

Maximal joint torque per agonist muscle size is considered as the torque‐producing capacity (Lieber & Friden, 2000). This capacity is often associated with various factors, including biological (e.g., gene polymorphism: Vincent et al., 2007), physiological (e.g., muscle fiber composition: Larsson & Moss, 1993), neurological (e.g., muscle neural activation; Klein, Rice, & Marsh, 2001; Trezise et al., 2016), and morphological factors (e.g., muscle fascicle length and pennation angle: Blazevich et al., 2009; Trezise et al., 2016). Although it remains controversial whether torque‐producing capacity is different among some populations related to aging (e.g., young vs. older individuals) and training status (e.g., trained vs. untrained participants) (e.g., Fukunaga et al., 2001), our previous study determined that subjects having the shortest and longest knee extensor MAs among adults were 39.1 mm and 49.1 mm, respectively, which differs by 10 mm (Miyake et al., 2017). Furthermore, Blazevich et al. (2009) reported that the knee extensor MA was different by about 15 mm among adults. These findings suggest that the joint MA dimension varies in a single population. Because of the close relationship between joint torque production and MA dimension, we hypothesized that greater joint MA would contribute to higher torque‐producing capacity in a single population. However, this hypothesis remains untested in various populations, including children. To determine our hypotheses, we examined the relationships between MA dimension and isometric and isokinetic torques of the knee extensors in preadolescent children. Thereafter, we examined the relationship between MA dimension and torque‐producing capacities during isometric and isokinetic contractions of the knee extensors in this population.

## 2 METHODS

### 2.1 Participants

Twenty boys (age: 10.7 ± 0.9 years; body height: 142.0 ± 8.1 cm; body mass: 34.7 ± 6.4 kg) volunteered to participate in this study. The participants were not involved in any specific sport training. Their maturity status was evaluated using years from peak height velocity assessed using existing regression equation (Mirwald, Baxter‐Jones, Bailey, & Beunen, 2002). The participants were at the age of pre‐peak height velocity (−3.2 ± 0.8 years). None of the participants had contraindications to magnetic resonance imaging (MRI). All participants and their parents were informed of experimental procedures and provided written consent to participate in this study. The study was approved by the Ethics Committee of Ritsumeikan University (BKC‐IRB‐2015‐019) and conducted according to the Declaration of Helsinki.

### 2.2 MRI measurement

**FIGURE 1:**
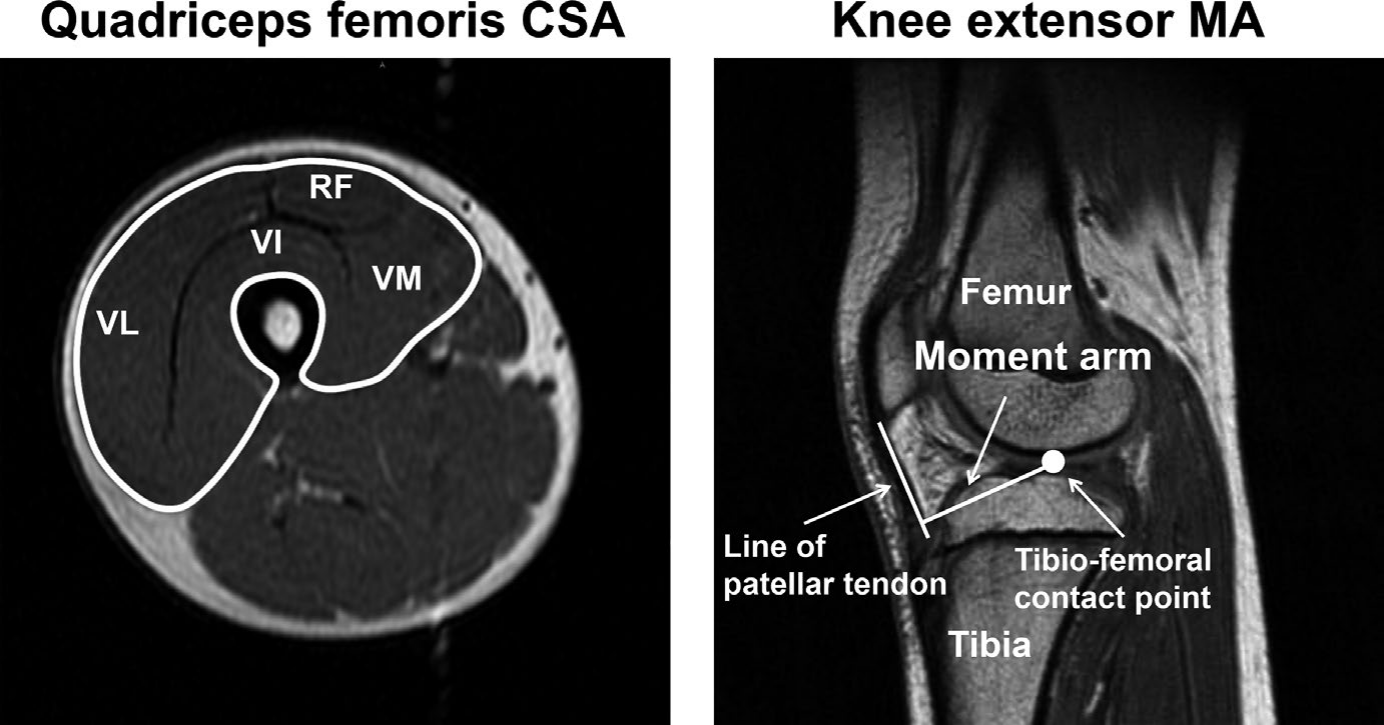
Representative magnetic resonance imaging scans for measuring the quadriceps femoris muscle volume (MV) and knee extensor moment arm (MA). The mid‐thigh quadriceps femoris cross‐sectional are (CSA) obtained at 50% of the thigh length. The quadriceps femoris muscle included rectus femoris (RF), vastus intermedius (VI), vastus lateralis (VL), and vastus medialis (VM). The quadriceps femoris MV was calculated by multiplying the sum of the CSAs along their length at intervals of 10 mm. The knee extensor MA was calculated as the distance between tibio‐femoral contact point and mid‐line of the patellar tendon

The representative images for calculating the quadriceps femoris muscle volume (MV) and knee extensor MA are presented in Figure 1. The MRI measurement was performed using a 1.5‐T magnetic resonance system (Signa HDxt; GE Medical Systems). Participants were placed in a supine position on the scanner bed, with both knees fully extended and both ankles set at the neutral position (i.e., 0°). The MRI analyses of the quadriceps femoris MV and knee extensor MA were performed using the same methods as our previous studies (Miyake et al., 2017; Tomita et al., 2018).

To measure the quadriceps femoris MV, axial T_1‐_weighted MRI scans of the thigh of the right leg were acquired with a standard body coil. Axial scans were obtained in successive slices with an inter distance of 10 mm from the inferior aspect of the greater trochanter to the lower edge of the femur with a repetition time of 600 ms, echo time of 7.6 ms, field of view of 480 mm, and matrix size of 512 × 256 pixels. The quadriceps femoris MV was calculated by multiplying the sum of cross‐sectional areas (CSA; CSAs) along their length at intervals of 10 mm.

To measure the knee extensor MA, three‐dimensional isotopic T_1_‐weighted MRI scans of the knee joint of the right leg were acquired with a 4 channels coil. Sagittal scans were obtained in successive slices with an inter distance of 1.2 mm with a repetition time of 11.3 ms, echo time of 5.1 ms, field of view of 280 mm, and matrix size of 256 × 256 pixels. The knee extensor MA was calculated as a distance between the tibio‐femoral contact point and the mid‐line of the patellar tendon. The knee extensor MA was calculated twice, and the mean of the two values was used for analysis.

The analyses for measuring the quadriceps femoris size and knee extensor MA were performed by the same investigator using image analysis software (OsiriX Version 5.6, Switzerland). The coefficient of variations (CV) of the measured two values of the mid‐thigh (i.e., 50% of the thigh length) quadriceps femoris CSA and knee extensor MA were 1.8% ± 0.5% and 0.8% ± 0.5% respectively. The intraclass correlation coefficients (ICC) of the two measurements were 0.979 and 0.981 respectively. The reproducibility of measurements of the quadriceps femoris size and knee extensor MA on two separate days has been reported in our previous studies (Miyake et al., 2017; Tomita et al., 2018).

### 2.3 Knee extensor torque and torque‐producing capacity

The knee extensor isometric and isokinetic torques of the right leg were measured using an isokinetic dynamometer (BIODEX system 3; BIODEX Medical). The participants were secured at the hip by a seat belt to prevent any movement of the hip joint. The hip and knee joint angles were fixed at 100° and 90° (full extension was at 180°) respectively. The ankle joint was attached to a bar connected to the force transducer. A 90° knee angle at the isometric torque measurement was selected based on previous studies (Blazevich et al., 2009; Tanaka et al., 2016; Trezise et al., 2016). Isokinetic torque measurements were performed at slow and fast angular velocities of 60°/s and 180°/s, respectively, through a 90° to 170° range of motion of the knee joint. Both angular velocities during the isokinetic torque measurements were selected based on previous studies (Grbic et al., 2017; Hoshikawa et al., 2009). Prior to the isometric and isokinetic torque measurements, participants practiced three submaximal trials under each condition prior to performing two maximal trials. After the practice was completed, the isometric trials were performed for 5 s each with a 1‐min rest period. The isokinetic trials were performed consecutively with three repetitions each per trial at both angular velocities for 60°/s and 180°/s. A 1‐min rest period was set as the rest interval between the two isokinetic angular velocity measurements. During these measurements, to elevate the participant's motivation, verbal encouragement was given by the examiners. Additionally, the real‐time torque data were displayed on a computer screen in front of the participant during maximal trials. The highest value for each measurement was used as for analyses. To assess the knee extensor torque‐producing capacity, it was calculated the knee extensor isometric or isokinetic torque per the quadriceps femoris MV.

The CVs of the highest and second highest values for the three torque measurement of all subjects were 3.9% ± 2.9% for isometric torque, and 5.2% ± 4.4% and 2.8% ± 1.8% for isokinetic torques at 60°/s and 180°/s respectively. Additionally, to assess the reproducibility of the three torque measurements, we measured the isometric torque and isokinetic torques at 60°/s and 180°/s on two separate days in 13 children (10.6 ± 0.8 years). The ICCs of heights for each day were 0.975 for isometric torque, and 0.993 and 0.982 for isokinetic torques at 60°/s and 180°/s respectively.

### 2.4 Statistical analysis

All data are presented as the mean ± *SD*. The Pearson correlation coefficient was used to assess the relationships between measured variables. To examine the relationships of the quadriceps femoris MV and knee extensor MA with knee extensor isometric torque and isokinetic torques, partial correlation analyses were performed after controlling for confounding factors. Model 1 and 2 were adjusted for body height and body mass respectively. Model 3 was adjusted for both body height and body mass. Stepwise multiple regression analyses were performed with physical size (i.e., body height and body mass) and knee extensor morphological (i.e., the quadriceps femoris MV and knee extensor MA) variables to explore the predictive variables for isometric and isokinetic torques. Statistical significance level was defined at *p* < .05. All statistical analyses were conducted using the IBM SPSS software (version 24.0; International Business Machines Corp).

## 3 RESULTS

Mean values of measured variables are summarized in Table 1. The quadriceps femoris MV correlated significantly with body height and body mass (*r* = 0.797 and 0.705, respectively; *p* < .001 for both). The knee extensor MA also correlated significantly with body height and body mass (*r* = 0.784 and 0.595, respectively, *p* < .01 for both). Furthermore, all three knee extensor isometric and isokinetic torques correlated significantly with body height (*r* = 0.680 to 0.817, *p* < .001 for all) and body mass (*r* = 0.706 to 0.790, *p* < .001 for all). Additionally, there was a significant correlation between the quadriceps femoris MV and knee extensor MA (*r* = 0.594, *p* = .006).

**TABLE 1:** Mean values of measured variables

	Mean ± *SD*
Body height, cm	142.0 ± 8.1
Body mass, kg	34.7 ± 6.4
Quadriceps femoris MV	699.2 ± 158.4
Knee extensor MA, mm	34.3 ± 2.4
Knee extensor torque	
Isometric torque, Nm	74.5 ± 26.6
Isokinetic torque at 60°/s, Nm	59.9 ± 23.0
Isokinetic torque at 180°/s, Nm	43.4 ± 14.4
Knee extensor torque producing capacity	
Isometric torque, kNm/cm^3^	106.3 ± 29.6
Isokinetic torque at 60°/s, kNm/cm^3^	84.6 ± 22.4
Isokinetic torque at 180°/s, kNm/cm^3^	61.6 ± 12.4

Abbreviations: MA, moment arm; MV, muscle volume.

Coefficient correlations between morphological variables and isometric and isokinetic joint torques in the knee extensors are shown in Table 2. The quadriceps femoris MV correlated significantly with all three knee extensor isometric and isokinetic torques (*r* = 0.617 to 0.804, *p* = .01 for all). The knee extensor MA also correlated significantly with the three knee extensor isometric and isokinetic torques (*r* = 0.701 to 0.806, *p* ≤ .001 for all).

**TABLE 2:** Correlation coefficients between morphological variables and isometric and isokinetic torques in the knee extensors

	Isometric contraction		Isokinetic contraction at 60°/s		Isokinetic contraction at 180°/s	
	*r*	*p* value	*r*	*p* value	*r*	*p* value
Quadriceps femoris MV	0.617	.004	0.766	<.001	0.804	<.001
Knee extensor MA	0.724	<.001	0.806	<.001	0.701	.001

The results of partial correlation analyses are shown in Table 3. For model 1 (i.e., adjusted for body height), only a significant partial correlation was observed between the knee extensor MA and isometric torque (partial *r* = 0.465, *p* = .045). For model 2 (i.e., adjusted for body mass), significant partial correlations were observed between the quadriceps femoris MV and isokinetic torques at 60°/s and 180°/s (partial *r* = 0.478 and 0.568, respectively, *p* < .05 for both). Such significant partial correlations were also observed between the knee extensor MA and all three isometric and isokinetic torques (partial *r* = 0.467 to 0.676, respectively, *p* < .05 for all). For model 3 (i.e., adjusted for both body height and body mass), only a significant partial correlation was observed between the knee extensor MA and isometric torque (partial *r* = 0.504, *p* = .033).

**TABLE 3:** Partial correlation analyses

		Isometric contraction		Isokinetic contraction at 60°/s		Isokinetic contraction at 180°/s	
		Partial *r*	*p* value	Partial *r*	*p* value	Partial *r*	*p* value
Model 1	Quadriceps femoris MV	0.169	.489	0.305	.205	0.439	.060
	Knee extensor MA	0.420	.073	**0.465**	**.045**	0.169	.490
Model 2	Quadriceps femoris MV	0.237	.329	**0.478**	**.038**	**0.568**	**.011**
	Knee extensor MA	**0.533**	**.019**	**0.676**	**.001**	**0.467**	**.044**
Model 3	Quadriceps femoris MV	0.084	.739	0.241	.335	0.382	.117
	Knee extensor MA	0.464	.053	**0.504**	**.033**	0.116	.436

Note

Model 1 was adjusted for body height. Model 2 was adjusted for body weight. Model 3 was adjusted for body height and body weight. Bold values indicate significant correlations (*p* < .05) between morphological variables and joint torque in knee extensors.

The results of stepwise multiple regression analyses are shown in Table 4. The knee extensor MA (*β* = 0.472, *p* = .018) and body mass (*β* = 0.425, *p* = .031) were the predictive variables for the knee extensor isometric torque (adjusted *R* ^2^ = 0.601, *p* < .001). The body height (*β* = 0.472, *p* = .031) and knee extensor MA (*β* = 0.436, *p* = .044) were predictive variables for the knee extensor isokinetic torque at 60°/s (adjusted *R* ^2^ = 0.706, *p* < .001). The body height (*β* = 0.649, *p* < .001) was only a predictive variable for the knee extensor isokinetic torque at 180°/s (adjusted *R* ^2^ = 0.649, *p* < .001).

**TABLE 4:** Multiple regression analyses

Dependent variable	Independent variable	Adjusted *R* ^2^	*β*	*p* value
Isometric torque	Knee extensor MA	0.601	0.472	.018
	Body mass		0.425	.031
Isokinetic torque at 60°/s	Body height	0.706	0.472	.031
	Knee extensor MA		0.436	.044
Isokinetic torque at 180°/s	Body height	0.649	0.817	<.001

**FIGURE 2:**
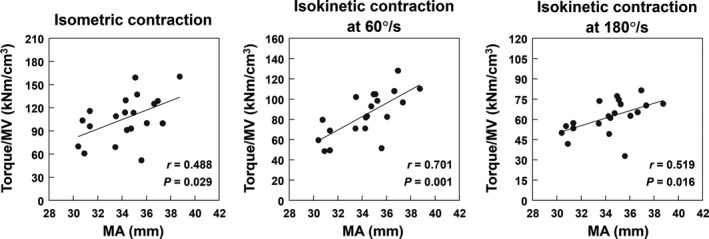
Relationships between MA and torque‐producing capacities during isometric and isokinetic contractions in the knee extensors. Torque‐producing capacity was calculated as the knee extensor isometric or isokinetic torque per the quadriceps femoris MV

Relationship between MA dimension and torque‐producing capacity in the knee extensors are presented in Figure 2. The knee extensor MA correlated significantly with all three torque‐producing capacities during knee extensor isometric and isokinetic contractions.

## 4 DISCUSSION

This study demonstrated that greater knee extensor MA in addition to quadriceps femoris MV correlated with higher torques during all three knee extensor isometric and isokinetic contractions. Moreover, even if there were adjusted for physical size variables (i.e., body height and/or body mass), some significant partial correlations were observed between MA dimension and isometric and isokinetic torques in the knee extensors. Furthermore, in multiple regression analyses with physical size and knee extensor morphological variables, the knee extensor MA was predictive variable for the knee extensor isometric and isokinetic torques at 60°/s. Additionally, greater knee extensor MA correlated with higher torque‐producing capacities during all three knee extensor isometric and isokinetic contractions. These findings suggest that the magnitude of joint MA dimension may play an important role in achieving superior torque production of the knee extensors in preadolescent boys.

This study also determined that the knee extensor MA correlated with the quadriceps femoris MV in children. We and others previously reported a positive relationship between MA dimension and muscle size of the knee extensors and plantar flexors in adults (Baxter & Piazza, 2014; Miyake et al., 2017; Tomita et al., 2018). In a previous study, O'Brien, Reeves, Baltzopoulos, Jones, and Maganaris (2009) examined the relationships of the knee extensor MA with physical and anthropometric variables in children. They demonstrated that knee extensor MA correlated with some physical (i.e., body height and body mass) and anthropometric (e.g., leg length) variables. However, their study did not examine the relationship between joint MA dimension and muscle size in children. In another study, Waugh, Blazevich, Fath, and Korff (2011) demonstrated that the plantar flexor MA correlated with lower leg circumference, which slightly reflects muscle size (Rolland et al., 2003). However, their study also did not examine a direct relationship between joint MA dimension and muscle size. Therefore, the present study is the first to determine the positive relationship between joint MA dimension and muscle size in children.

Several studies reported a positive relationship between MA dimension and joint torque of the knee extensors and planter flexors in adults (Baxter & Piazza, 2014; Blazevich et al., 2009; Trezise et al., 2016). However, to the best of our knowledge, no study has examined this relationship in children. Thus, the present study is the first to determine the positive relationship between MA dimension and joint torque in children. In a previous study, Blazevich et al. (2009) demonstrated that the knee extensor MA correlated moderately with knee extensor isometric torque but not isokinetic torques at both slow and fast angular velocities in adults. In contrast, the present study demonstrated that knee extensor MA correlated strongly with isometric torque and slow and fast isokinetic torques in children. Considering the findings of these present and previous studies, the contribution of MA on joint torque production, particularly during isokinetic torque, may be higher in children than in adults. Previous studies reported that neural voluntary activation during maximal contraction may be lower in children than in adults (Kluka et al., 2015; O'Brien et al., 2010). This finding may contribute to understand a higher contribution of MA on joint torque production in children than that in adults. Therefore, we suggest that greater MA in children may be a more important factor for producing higher joint torque than that in adults.

In this study, stepwise multiple regression analyses revealed that knee extensor MA was a predictive variable for the knee extensor isometric and slow (i.e., 60°/s) isokinetic torques. By contrast, the knee extensor MA was not selected as predictive variable for fast (i.e., 180°/s) isokinetic torque because body height was only a predictive variable for this torque. Baxter and Piazza (2014) suggested that smaller rather than greater joint MA may have a positive effect for higher torque production during fast isokinetic contraction in the plantar flexors. Considering the findings of the present and previous studies, contribution of MA on joint torque production during fast isokinetic contraction may be less than that during isometric and slow isokinetic contractions. Blazevich et al. (2009) determined that the fascicle lengths of the quadriceps femoris muscles were included in predictive variables for the knee extensor isokinetic torque during the fast contraction (i.e., 300°/s). The longer fascicle length is related to higher muscle contractile speed (Kumagai et al., 2000). Therefore, compared to joint MA dimension, other morphological factors, such as the fascicle length, may be more important for achieving superior torque production during fast isokinetic contraction.

We and others previously determined that MA dimension in several joints varied among participants (i.e., adults) in a single population (Baxter & Piazza, 2014; Blazevich et al., 2009; Miyake et al., 2017; Tomita et al., 2018). Because the magnitude of joint torque is morphologically determined as the product of muscle size and MA dimension, subjects having longer MA can theoretically have higher torque‐producing capacity despite a given muscle size than subjects having a shorter MA. Thus, prior to the present study, we hypothesized that greater MA would relate to higher torque‐producing capacity in the knee extensors of children. As expected, we demonstrated the positive relationships between MA dimension and torque‐producing capacities during all isometric and isokinetic contractions in the knee extensors of preadolescent boys. To the best of our knowledge, no study has found this relationship in various populations, including children. Therefore, for the first time, we suggest that greater MA may be an advantageous morphological factor in achieving higher torque‐producing capacity.

The present study has a major limitation. In the present study, we measured the knee extensor MA with both knees fully extended because of a technical limitation of our MRI measurement. Thus, the joint angle (i.e., 180°) measured during the knee extensor MA is different from that during isometric (i.e., 90°) and during isokinetic (90° to 170°) contractions. Nevertheless, previous studies reported that the difference in the full‐extended knee extensor MA between populations remained constant throughout the full range of motion of the knee joint (Krevolin, Pandy, & Pearce, 2004; Tsaopoulos, Baltzopoulos, & Maganaris, 2006). Therefore, we have considered that the knee extensor MA measured in the present study can reflect the difference in MA dimensions throughout the range of motion during isometric and isokinetic contractions among participants of a single population.

Another limitation of the present study was that, although we determined a positive relationship between MA dimension and torque‐producing capacity of the knee extensors, we could not measure some important parameters for torque‐producing capacity. The torque‐producing capacity is often associated with various factors (Blazevich et al., 2009; Klein et al., 2001; Larsson & Moss, 1993; Trezise et al., 2016; Vincent et al., 2007). Of those, morphological factors, such as pennation angle and muscle fascicle length may affect torque‐producing capacity (Blazevich et al., 2009; Trezise et al., 2016). Furthermore, neuromuscular factors, such as neural voluntary activation may play important roles in determining the torque‐producing capacity (Trezise et al., 2016). However, in this study, we did not measure some important morphological and neuromuscular parameters for regulating joint torque productions, including torque‐producing capacity. To clearly understand the impact of MA dimension on torque‐producing capacity, further studies are needed to examine the individual and interaction effects of various factors on torque‐producing capacity.

In a clinical implication, the findings of this study propose that children having a longer MA can have a more enhanced toque production despite a given muscle size than children having a shorter MA. As is well known, the magnitude of joint torque production is related to superior athletic performances in various athletes (Alexander, 1989; Hoshikawa et al., 2010; Sliwowski et al., 2017). In a recent study, we determined that greater knee extensor MA correlated with higher sprint performance in adult sprinters (Miyake et al., 2017; Tomita et al., 2018). Additionally, we and others demonstrated that larger quadriceps femoris size correlated with higher sprint performance in preadolescent boys (Enomoto, Oda, & Kaga, 2019; Tottori et al., 2018); thus, that the magnitude of the knee extensor torque production may play an important role for achieving superior sprint performance in this young population. Based on the findings of our studies, children having a greater knee extensor MA may have potential for successful athletes, especially sprinters, potentially by enhancing the knee extensor torque, than children having a smaller knee extensor MA. Therefore, information from the present study may be helpful in preselecting/selecting suitable sports/events and understanding individual features for children.

## 5 CONCLUSION

This study demonstrated that greater MA correlated with higher isometric and isokinetic joint torques of the knee extensors in preadolescent boys. Furthermore, we found that greater MA correlated with higher torque‐producing capacities, assessed as the joint torque per the quadriceps femoris MV, during isometric and isokinetic contractions of the knee extensors in this population. This study is the first to determine that the joint MA dimension may play an important role in achieving superior torque production in children.

## CONFLICT OF INTEREST

The authors declare no conflict of interest.

## AUTHOR CONTRIBUTIONS

N.T., T.S., T.I., and S.F. designed the experiment. N.T., T.S., and M.H. performed experiments. N.T., T.S., and M.H. analyzed data and interpreted results of experiments. N.T. and T.S. wrote the manuscript. T.I. and S.F. edited and revised manuscript. All authors read and approved the final manuscript.
